# Reduced Birth Weight and Exposure to Per- and Polyfluoroalkyl Substances: A Review of Possible Underlying Mechanisms Using the AOP-HelpFinder

**DOI:** 10.3390/toxics10110684

**Published:** 2022-11-12

**Authors:** Claudia Gundacker, Karine Audouze, Raimund Widhalm, Sebastian Granitzer, Martin Forsthuber, Florence Jornod, Maria Wielsøe, Manhai Long, Thórhallur Ingi Halldórsson, Maria Uhl, Eva Cecilie Bonefeld-Jørgensen

**Affiliations:** 1 Institute of Medical Genetics, Medical University of Vienna, 1090 Vienna, Austria; 2 Unit T3S, Université Paris Cité, Inserm U1124, 75006 Paris, France; 3 Department of Public Health, Aarhus University, 8000 Aarhus, Denmark; 4 Faculty of Food Science and Nutrition, University of Iceland, 102 Reykjavík, Iceland; 5 Department of Epidemiology Research, Statens Serum Institut, 2300 Copenhagen, Denmark; 6 Environment Agency Austria, 1090 Vienna, Austria; 7 Greenland Center for Health Research, Greenland University, Nuuk 3905, Greenland

**Keywords:** fetal growth, PFOS, PFOA, PFHxS, PFNA, PFDA

## Abstract

Prenatal exposure to per- and polyfluorinated substances (PFAS) may impair fetal growth. Our knowledge of the underlying mechanisms is incomplete. We used the Adverse Outcome Pathway (AOP)-helpFinder tool to search *PubMed* for studies published until March 2021 that examined PFAS exposure in relation to birth weight, oxidative stress, hormones/hormone receptors, or growth signaling pathways. Of these 1880 articles, 106 experimental studies remained after abstract screening. One clear finding is that PFAS are associated with oxidative stress in in vivo animal studies and in vitro studies. It appears that PFAS-induced reactive-oxygen species (ROS) generation triggers increased peroxisome proliferator-activated receptor (PPAR)γ expression and activation of growth signaling pathways, leading to hyperdifferentiation of pre-adipocytes. Fewer proliferating pre-adipocytes result in lower adipose tissue weight and in this way may reduce birth weight. PFAS may also impair fetal growth through endocrine effects. Estrogenic effects have been noted in in vivo and in vitro studies. Overall, data suggest thyroid-damaging effects of PFAS affecting thyroid hormones, thyroid hormone gene expression, and histology that are associated in animal studies with decreased body and organ weight. The effects of PFAS on the complex relationships between oxidative stress, endocrine system function, adipogenesis, and fetal growth should be further explored.

## 1. Introduction

Birth weight is a widely studied outcome in environmental health studies because it is an important predictor of neonatal health, easily and accurately measured, and sensitive to toxic effects [1,2].

By definition, the term “low birth weight” refers to a weight <2500 g [3]. Small for gestational age (SGA) describes newborns with a birth weight at least two standard deviations below the mean for gestational age in relation to a reference population [4]. This definition does not necessarily correspond to the more common one that classifies SGA as birth weight below the tenth percentile of gestational age [4,5]. The prevalence of SGA births is 10–12% in U.S., Chinese, and European populations [6,7,8], but can deviate significantly up to a prevalence of 42% in term SGA infants, for example, in South Asia [9]. According to Ludvigsson et al. [5], SGA occurs in more than 30 million infants each year and is associated with an increased risk of stillbirth, neonatal mortality, and death in infancy.

About 70% of the total variation in birth weight is explained by genetics, and the remaining variance is attributed to the environment, including pollution [4,9,10,11]. In a prospective cohort of dichorionic twin births, the contribution of fetal genetics to estimated fetal weight (EFW) peaked at 71% in the second trimester, whereas shared environment explained most of the phenotypic variation in fetal growth in the first trimester (54% contribution to EFW) [12].

The maternal factors associated with lower birth size include maternal birth weight, maternal height and age, previous stillbirth, preterm birth, and SGA birth, parity (as nulliparous women have higher risk for delivering SGA births), low socioeconomic status, smoking, drug consumption, and diseases such as infections and anemia. Together, maternal and paternal body size contribute to 3–12% of the variation in birth weight. Paternal factors, in general, are less well-studied [4].

The key regulators of fetal growth are fetal insulin and insulin-like growth factor (IGF)1 and IGF2 with their binding proteins and receptors, which modulate the action of IGF1 and 2 [4,13,14]. IGF1 regulates size prenatally and postnatally until adolescence, whereas maternally imprinted (paternally expressed) IGF2 is primarily a growth factor for fetal growth [14]. The placenta mediates maternal and fetal oxygen and nutrient exchange and has endocrine functions, thereby having a significant impact on birth size [15,16,17]. The levels of placental growth hormone variant (GHV) and placental growth factor are significantly associated with placental and fetal growth [4,18,19]. Numerous pathological conditions, including vascular impairment of the placenta, can result in SGA births [20,21]. 

Pollutants that may affect fetal growth include endocrine disrupting per- and polyfluoroalkyl substances (PFAS) [22]. PFAS are industrial chemicals with very stable C-F bonds in the perfluoroalkyl moiety, which allows an exceptionally wide range of applications but also results in high persistence [23,24]. Perfluoroalkane sulfonic acids (PFSAs) and perfluoroalkyl carboxylic acids (PFCAs) are particularly persistent and bioaccumulate in wildlife and humans [24]. Human exposure occurs mainly through food and drinking water and, to a lesser extent through inhalation and dermal uptake [23,25]. Prenatal exposure is of high concern because it can affect fetal growth [24,26,27] and has therefore been the subject of numerous epidemiological studies. Mostly, fetal growth has been analyzed as a continuous outcome (birth weight, birth length, head circumference, ponderal index) and less frequently as SGA or birth weight Z-score, another weight-at-age index. Existing studies have been systematically reviewed and, to some extent, reanalyzed in several papers [6,28,29,30,31,32,33].

As summarized in Table 1, most analyses confirmed that prenatal exposure to perfluorooctanoic acid (PFOA) or perfluorooctane sulfonic acid (PFOS) is inversely associated with birth weight. In addition, Fan et al. [33] estimated the number of PFOA-associated low-birth-weight cases to be 461,635 (95% confidence interval: 57,418 to 854,645) in the last two decades, mainly in Asian regions. Yet, the observed decrements in birth weight were, on average, small and within the normal range of distribution, so they may have little or no direct effect on infant morbidity or mortality [1]. Accordingly, most authors [28,29,30,31,32] expressed that the significance of modest reductions in birth weight remains unclear. Govarts et al. [6] pooled data from seven European birth cohorts and found significantly higher SGA risk for cases with high PFOA exposure levels (OR: 1.64). Regarding PFOS, an increased SGA risk was present in cases where mothers had smoked during pregnancy (OR: 1.63); similar effects were reported by Rokoff et al. [34]. Higher odds of SGA birth were also found regarding PFOA (OR: 1.20) and PFNA (OR: 1.32) exposure in African American women [35].

Most studies examine the effect of a single compound on fetal growth. However, pregnant women are exposed to a multitude of chemicals/stressors, including PFAS mixtures. Rokoff et al. [34] found concomitant prenatal exposure to maternal smoking, residential black carbon, and PFOS to be additively associated with lower birth weight z-scores. Exposure to a mixture of endocrine disrupting chemicals, i.e., PFAS, triclosan, phthalates, non-phthalate plasticizers, bisphenols, polycyclic aromatic hydrocarbons, pesticides, and polychlorinated biphenyls (PCBs), has been shown to be associated with lower birth weight z-scores and slower infant growth spurt rate, particularly in girls [22]. Both exposure to a mixture of endocrine disrupting chemicals including PFAS or to actual serum mixture of PFAS has been shown to be associated with lower birth weight [22,36].

Prenatal PFAS exposures are also associated with other adverse pregnancy outcome, such as, for instance, PFOA and PFOS with late-onset preeclampsia [37]. This adds to the complexity of the situation, as the pathophysiology of preeclampsia, which particularly affects first-time pregnancies and is often associated with fetal growth restriction, is insufficiently understood [38].

The many uncertainties in the observations on prenatal PFAS exposure and fetal growth raise the fundamental question of causality of the association. Several PFAS-related mechanisms have been proposed, including effects on the IGF axis [39], reduced blood vessel formation [40], and disturbed placental development and physiology that change placental weight [41,42,43] and placental endocrine function [44].

Overall, our knowledge of PFAS-induced mechanisms causing or contributing to reduced fetal growth is incomplete. One goal of the Human Biomonitoring Initiative for Europe (HBM4EU [45]) was to integrate data on mechanistic toxicology, human biomonitoring, and adverse outcome pathways (AOPs) to support human health risk assessment. As part of the HBM4EU project, our aim was to systematically search for PFAS-induced toxicity mechanisms. Using the AOP-helpFinder tool [46] we aimed to identify studies that investigated PFAS-related effects on hormones and hormone receptors, nutrients, oxidative stress, and growth signaling pathways. Based on the identified relevant experimental studies, we manually analyzed for modes of action (MoA), and if the mechanisms could be linked in a plausible way, these were used to propose new AOPs.

## 2. Materials and Methods

A combined literature and manual curation approach was conducted to rapidly identify and collect existing published and dispersed information on low birth weight and PFAS exposure to gain a better understanding of their MoA and to inform the development of future AOPs.

### 2.1. Development of the Search Term Lists

The first step was to create two lists in order to be able to run the AOP-helpFinder. First, a list of MeSH terms (a controlled vocabulary of the U.S. National Library of Medicines) and other free-text search terms related to the studied outcomes was compiled (Table 2). These search terms were complemented by a list of matching molecular initiating events (MIEs) and key events (KEs), which were selected by experts (Table 2). Then, a third list related to the PFAS compounds and their synonyms was generated, which includes five PFAS compounds (Table 3).

### 2.2. Running the AOP-helpFinder Tool

The full available literature (>33 millions of publications) in the *PubMed* database (accessed March 2021) was used for the screening. First, all publications related to at least one of the PFAS compounds were identified and kept for the text mining. Then, the AOP-helpFinder was applied, which is a tool based on artificial intelligence [47,48] that was previously successfully applied to develop new AOP [49,50,51]. This tool allows to automatically identify co-occurrence between terms from both lists, i.e., between an outcome search term and a PFAS, in published abstracts was run. Then, we kept only abstracts co-mentioning at least one outcome search term and one PFAS. Default parameters were used (i.e., screening the full abstracts and calculation of the two scoring systems to identify as much as possible the relevant associations) [52].

### 2.3. Manual Curation

We excluded abstracts for further investigation that were in duplicate or not significant for the proposed study focus. Identified abstracts co-mentioning outcome search terms and PFAS were manually investigated, and when necessary, the full publications were read to confirm the linkage and help building the AOPs.

### 2.4. Flowchart

**Figure 1:**
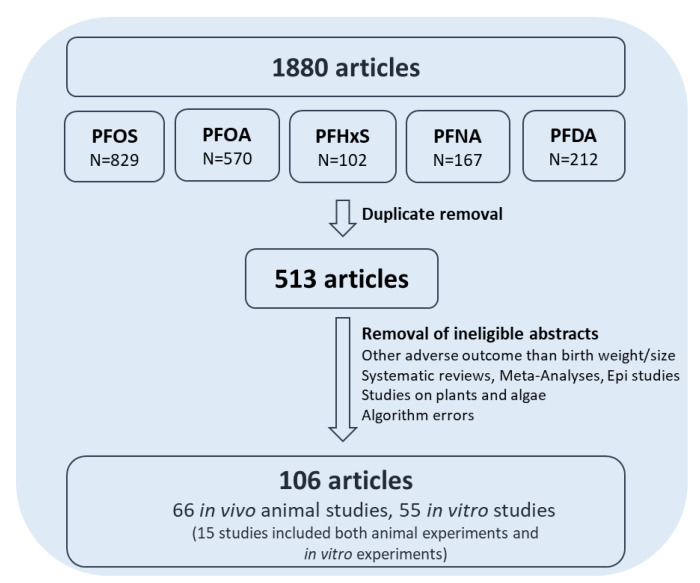
The sequence of the AOP-helpFinder search.

Overall, 1880 abstracts were retrieved and reviewed for eligibility and significance. After exclusion of ineligible abstracts and abstract screening, 106 experimental studies remained (Figure 1). The experimental studies were searched for molecular initiating events (MIEs) and key events (KEs) (see Table 4, Table 5, Table 6, Table 7, Tables 8–11 for specific references).

## 3. Results

Exposure to PFAS and associated outcomes was investigated in a variety of experimental in vivo and in vitro studies (Table 4, Table 5, Table 6, Table 7, Tables 8–11). The reviewed studies encompass a large variety of different species used as in vivo models (fish, mollusks, crustacea, insects, nematodes, amphibians, and rodents) as well as in vitro models (yeast, fish, amphibian, rodent, monkey, and human cells). Most studies focused on PFOA or PFOS exposure, while fewer studies also included perfluorononanoic acid (PFNA), perfluorohexanesulfonic acid (PFHxS), or perfluorodecanoic acid (PFDA). Mixture exposure were also studied in a small number of in vivo and in vitro studies (Table 5, Tables 8 and 9). The PFAS treatments used in these studies can only approximate the human exposure situation. Often, superphysiological concentrations were tested over short exposure times ranging from hours/days in vitro to several weeks in vivo.

### 3.1. PFAS-Associated Cytotoxicity and Oxidative Stress

The in vivo studies demonstrate that PFOA and PFOS exposure can cause various outcomes, including reduced body weight and fetal and offspring weight, adverse effects on reproduction and development, changes in metabolism (i.e., altered levels of amino acids, lipids, and glucose), and changes in liver function and the (epi-)genome. The direction of these effects varied greatly (very often, effects in both directions were observed), so firm conclusions on these PFAS-induced adverse outcomes cannot be drawn (Table S1).

However, both PFOA and PFOS showed co-occurrence of oxidative stress and animal death or increased cellular damage in 3 in vivo and 13 in vitro studies (Table 4 and Table 5). Although the effects were observed to occur in a dose-dependent manner, the effect concentrations differed between the model of investigation. In general, the concentrations at which oxidative stress appeared were often lower than those at which cytotoxicity was induced, suggesting that oxidative stress may precede animal lethality or cell damage (Tables S1 and S2). Information on cell models, (animal) species, effect concentrations, and exposure route and time is given in Tables S1 and S2. In addition to PFOA and PFOS, the in vitro studies also showed consistent increase of oxidative stress upon PFNA, PFHxS, or PFDA exposure going along with increased cell damage (including reduced cell number, increased apoptosis, increased cytotoxicity, and decreased viability) after exposure to PFHxS. In the case of PFNA and PFDA, only a few studies have been conducted to investigate cell damage, and these do not provide a conclusive picture regarding the direction of the effect (Table 5). Possible reasons for this inconsistency are the different model systems (whole organism versus cell culture) and differences in treatment concentrations and exposure time.

### 3.2. PFAS-Associated Activation of PPAR, AKT, and MAPK Signaling Pathways

The here-reviewed in vivo and in vitro studies show that PFAS exposure resulted in an increased gene expression of transcription factors peroxisome proliferator-activated receptor (PPAR)α and PPARγ. In addition, PFAS were found to enhance the phosphorylation of key cellular signaling molecules including AKT, also known as Protein kinase B, and the mitogen-activated-protein kinases (MAPKs) ERK, JNK, and p38, thereby activating them (Table 6, Table 7, and Table S3). Information on (animal) species, cell models, effect concentration, and exposure time is given in Table S3. The aforementioned effects on gene expression and phosphorylation were independent of the PFAS compound used in the in vivo and in vitro studies (PFOA, PFOS, and PFHxS). However, there was an unexpected finding regarding tissue-specific response to PFOA exposure (Table 6). AKT activity reduced upon PFOA exposure in adipose tissue. This phenomenon was not found in liver and muscle tissue from the same mice, where AKT activity was actually increased after PFOA treatment.

### 3.3. PFAS-Associated Endocrine Effects

#### PFAS-Associated Estrogenic and Androgenic Effects

The in vivo studies generally found increased estrogen levels and decreased testosterone levels after PFAS exposure (Table 8 and Table S4). The effect doses ranged from 1–25 mg/kg/day in rodent and 25–250 µg/L in fish (Table S4). The animal species, exposure route, range, and time are given in Table S4. Increased estrogen receptor (ER) levels (RNA and/or protein) were also found in the majority of the here-reviewed in vivo studies, whereas the androgen receptor (AR) levels (RNA and/or protein) were decreased. The changes in hormone levels might relate to changes in the steroidogenesis cytochrome (CYP) enzyme levels, which were found altered in several studies. Many in vivo studies also measured the vitellogenin (VTG) protein and reported increased levels conforming the estrogenic activities of PFAS. Other estrogenic and androgenic effects found in the in vivo studies include altered sperm genesis, altered gene expression of the hypothalamic–pituitary–gonadal–liver (HPGL) axis, and reduced anogenital distances (AGD) and testicular weights in male offspring (Table 8 and Table S4). The results from the in vitro studies (Table 9 and Table S5) confirm the in vivo results. The specific cell model and exposure range and time are given in Table S5. The estrogen production was generally increased, and the testosterone production decreased after PFAS exposure. The majority of studies found agonistic estrogenic activities of the PFAS in the reporter gene assays; however, only one study found estrogenic effects with the E-screen assay. Of the six studies investigating androgenic receptor activities of PFAS, only one study [99] reported antagonistic effects. The H295R steroidogenesis assay or aromatase activity assay were used to investigate in vitro effect on the steroidogenesis, and the results were conflicting. Some found increased expression of the steroidogenesis CYP enzymes and decreased aromatase activity [99,100,101], while many others found no effect. Other estrogenic-related outcomes in the in vitro studies include altered expression of estrogen-responsive biomarker genes and increased progesterone and estrone level (Table 9 and Table S5).

Most in vivo studies showed that PFOS, PFHxS, and PFOA exposure affect the level of thyroid hormone (TH) by decreasing T4 (three increase, eight decrease), while T3 level varied in different studies (four increase, five decrease) (Table 10). The animal species, exposure route, range, and time are given in Table S6. The effect doses ranged from 1–63 mg/kg/day in rodent and 0.2–0.5 g/L in fish in regard to the thyroid effects (Table S6). Interestingly, in the three zebrafish PFAS exposure studies, the T3 level increased, whereas in rodent rat studies, the T3 levels decreased, but for the single mice study, the T3 increased. Two studies show, respectively, a PFOS-related decrease of TSH and thyroglobulin (TG), whereas another study observed an increase in TSH-receptor (TSHR) and thyroperoxidase (TPO). In addition, for thyroid cell histology, PFOS elicited a decrease in nuclear area in zebrafish embryos, and a PFOS substitute (F-538) caused thyroid follicular hyperplasia in adult female rats. Thus, the in vivo studies also observed that PFAS exposure could result in decreased embryo mass or pup birth weight and abnormal morphology in thyroid cell. The reviewed 18 studies reported that PFASs influences the expression of thyroid-hormones-related genes in zebra fish embryos, adult rats, amphibians/Xenopus laevis, pregnant mice, and chicken embryos (Table 10 and Table S6). 

For the in vitro studies, overall results are shown in Table 11, and the specific cell type, exposure range, and time are given in Table S7. Ten of the in vitro studies showed that PFAS (PFOS, PFOA, PFNA, PFHxS, and PFDA) bind to the human thyroid hormone transport protein transthyretin (TTR) although the binding potency was lower than TH (Table 11). Thus, the in vitro studies indicate that PFAS can interfere with TH transport in vivo by competitively displacing TH from TTR. PFAS also bind to TH receptors (TRα and TRβ) and activate their transcriptional activity and/or displace T3, causing a transcriptional decrease. In general, T-screen studies in rat Gh3 cells elicited that PFAS antagonized the T3-induced GH3 cell growth, whereas PFOS or its substitute exposure alone could increase cell growth. PFOS and PFOA increased the T4 level in rat hepatic cells. Some PFAS such as PFOS, PFOA, and PFHxS inhibit iodine uptake in both human and rodent cells. In human carcinoma cells, PFOS and PFOA inhibited TPO activity, an enzyme important for TH biosynthesis. PFOS elicited an altered steroidogenic gene expression in human H295R cells. Moreover, our review includes reports on PFAS molecular docking by fitting into the receptor pocket of thyroid receptors. Only two study evaluated effect of PFAS (PFOS, PFOA, PFNA, PFHxS, and PFDA) on thyroxine-binding globulin (TBG) and found no significant in vitro effect (Table 11,Table S7).

## 4. Discussion

**Figure 2:**
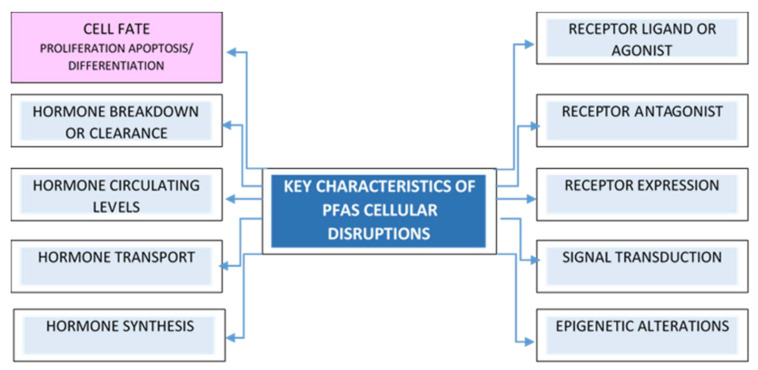
The key characteristics of potential PFAS cellular disruptions for hazard identification.

Exposure to endocrine-disrupting chemicals can affect maternal and fetal health, including long-term health effects later in life [159,160] (Figure 2). The underlying mechanisms are not yet well-understood. This review aimed to identify and further describe mechanisms that may underlie fetal growth reduction to better understand prenatal PFAS exposures and contribute to the establishment of potential new AOPs.

### 4.1. Experimental Studies on Oxidative Stress and Cytotoxicity

The dose-dependent lethality of PFAS might stem from cytotoxic properties that were found in in vitro studies for PFOA and PFOS but also for PFNA and PFHxS (Table 5). Although all these PFAS seem to be toxic to cells, the molecular mechanism behind this is unknown. It is not fully understood if and how PFAS enter a cell. Due to their amphiphilic structure, passive diffusion across a cellular membrane seems unlikely, indicating an active transport mechanism for this substance class [161]. It has been suggested that PFAS could be substrates for several transporter proteins, including organic anion transporters (OATs) and ATP-binding cassette (ABC) transporters [76,162,163]. In addition, PFAS could enter cells bound to protein ligands, such as albumin and fatty acid binding proteins [161].

PFAS-induced oxidative stress is a well-documented and likely mechanism explaining cytotoxicity (reviewed by [164]). However, it remains unknown whether PFAS directly generate oxidative stress or if PFAS-associated oxidative stress is an indirect effect.

Oxidative stress results from an imbalance between production and accumulation of oxidizing species (most importantly, reactive oxygen reactive species (ROS) such as hydroxyl radicals (•OH), superoxide radicals (O2•−), singlet oxygen (^1^O_2_), and hydrogen peroxide (H_2_O_2_)) in cells or tissues and the inability of a biological system to detoxify these reactive products. ROS are naturally produced in mitochondria and crucial mediators of many physiological processes. They become toxic, when present in excess, by oxidizing macromolecules such as DNA, proteins, and lipids. Therefore, cells have developed various antioxidant defensive mechanisms, including enzymes such as superoxide dismutase (SOD), catalase (CAT), and those constituting the glutathione system, to be protected from ROS-induced cellular damage [165,166].

Excessive ROS can activate different cellular signaling pathways, including the MAPKs JNK, ERK, and p38. MAPK signaling pathways have fundamental roles in the induction or inhibition of apoptosis. Constant ROS-mediated activation eventually leads to apoptosis [167]. Global induction of ROS-mediated apoptosis via PFAS is very unlikely to explain the relatively mild effects on birth weight, as the PFAS concentrations required to induce this apoptosis do not resemble the in vivo situation, as they are hyper-physiological.

It is possible that reduced birth weight is not the consequence of cell death but mass loss from reduced cell proliferation. One possibility to reduce cell proliferation is to differentiate cells (e.g., pre-adipocytes) into a non-dividing state (e.g., mature adipocytes) [168]. Adipogenesis, the maturation of adipocytes from adipose tissue derived mesenchymal stem cells, is an ROS-regulated differentiation process [169,170]. During adipogenesis, mesenchymal stem cells undergo a first differentiation step to pre-adipocytes, which still have proliferation potential. A second differentiation step turns pre-adipocytes into mature adipocytes, which no longer have the ability to proliferate [171].

During adipogenesis, AKT is activated by recruitment to the cell membrane, where it is phosphorylated by different proteins, e.g., phosphatidylinositol 3-kinase (PI3K) or mTORC2 or PDK1/2 [172,173]. Activated AKT increases the expression of transcription factor PPARγ, which enhances the expression of adipogenic genes [174]. In addition, MAPKs JNK, ERK, and p38 are activated during adipogenesis [174]. The universal activation mechanism of MAPKs is that MAPK-kinase-kinases (MAPKKKs) phosphorylate and activate MAPK-kinases (MAPKKs). These MAPKKs in turn activate the MAPKs (p38, JNK, and ERK) by phosphorylation [175].

From the studies reviewed here, both oxidative stress and activation of AKT and MAPK signaling increase in response to PFOA and PFOS exposure (Table 6, Table 7, and Table S3). Interestingly, a decrease in total body weight is accompanied by structural changes in adipose tissue [67] and a specific decrease in adipose tissue weight after PFOA treatment [75].

**Figure 3:**
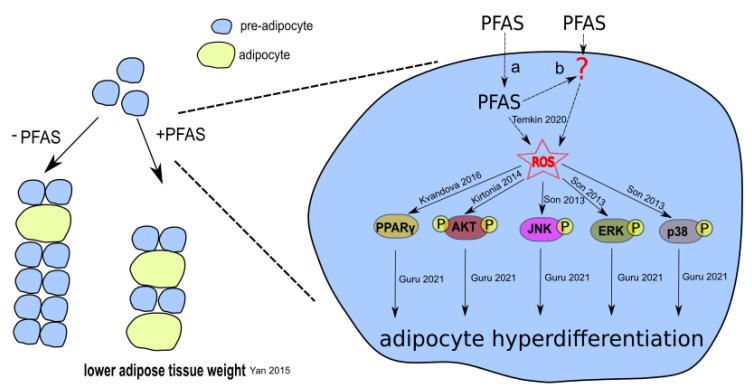
Possible mechanism of PFAS-associated reduction in adipose tissue. PFAS generate ROS directly (a) or indirectly (b). ROS trigger elevated PPARγ expression as well as activation of AKT, JNK, ERK, and p38 by phosphorylation, resulting in hyper-differentiation of pre-adipocytes. As a result, fewer proliferating pre-adipocytes remain after PFAS exposure, causing lower adipose tissue weight. Temkin 2020 [164]; Kvandova 2016 [177]; Kirtonia 2020 [176]; Son 2013 [178]; Guru 2021 [174]; Yan 2015 [75]. The proposed mechanism is not related to existing AOPs (AOP-Wiki).

Taken together, we propose a mechanism by which PFAS could lower adipose tissue weight (Figure 3) and thereby reduce birth weight. This model is based on several facts: (I) physiological PFAS concentrations are sufficient to generate ROS [164]; (II) ROS activate PPARγ, AKT, and MAPKs [176,177,178]; and (III) activation of these proteins is involved in adipogenesis [174].

It is conceivable that PFAS exposure could lead to differentiation of more pre-adipocytes to mature adipocytes. Indeed, it was demonstrated that PFOA, PFOS, PFHxS, and PFNA could differentiate the 3T3-L1 pre-adipocyte cell line into adipocytes in vitro [179].

The molecular mechanism could be PFAS-associated ROS production that triggers adipogenesis via increased expression of PPARγ-related genes and activation of MAPKs ERK, JNK, and p38 as well as AKT. Interestingly, AKT phosphorylation actually decreased and not increased (as expected during adipogenesis) in adipose tissue of PFOA-treated mice [67,75]. This could be caused by the insulin-resistant phenotype of these mice, as decreased AKT phosphorylation was described during insulin resistance [180,181]. Why AKT phosphorylation was specifically reduced in adipose tissue but not muscle or liver tissue should be clarified in future studies.

Nevertheless, the upregulation of some or all of these aforementioned pathways could lead to more differentiated adipocytes and fewer proliferating pre-adipocytes in response to PFAS. Fewer pre-adipocytes would over time lead to a reduced number of adipocytes and overall lesser adipose tissue weight. Thus, PFAS exposure would result in less adipose tissue mass and, in this way, may reduce birth weight. In studies on rodents, a decrease in adipose tissue mass after PFOS and PFOA treatment has already been found [75,182,183]. In women, an inverse relationship between PFOA, especially PFNA and PFDA, exposure and body fat mass was observed [184]. Whether a reduction in adipose tissue mass in response to PFAS exposure in general (or at least for PFOA, PFOS, PFHxS, PFNA, and PDFA) could result in lower birth weight is a matter for future studies. It is noteworthy that a prospective cohort showed a negative association between maternal PFOA and PFNA concentrations and adiposity at birth [185]. However, this effect could be age-dependent, as prenatal PFAS exposure has negative associations with body mass index in early life (first 2 years) and positive associations in childhood and adolescence [32]. However, no clear sex-specific differences were found, which may indicate the involvement of endocrine influences [186,187,188,189].

### 4.2. PFAS-Associated Endocrine Effects

A few reproductive and developmental toxicity studies have been conducted that were primarily focused on long-chain PFAS, including PFOS, PFOA, and PFNA in mice and rats [190]. In Sprague–Dawley rats, GenX (industrial replacement of PFOA) alters maternal and fetal glucose and lipid metabolism and produces neonatal mortality, low birthweight, and hepatomegaly [145]. Neonatal morbidity and mortality with exposure to high doses of PFAS and growth deficits and developmental delays were noted in offspring exposed to lower doses [190]. Lactation impairment was observed in mice [191], which led to an increased offspring mortality [192]. Studies have indicated a role of placental dysfunction in these adverse developmental outcomes [43]. Systematic reviews [190] support a relationship between in utero exposure to PFOA and PFOS and reduced fetal growth in animals and humans, and the relationship between PFOA and reduced fetal growth in mice was validated [43,193]. In addition, PFAS are reported to have reproductive effects such as ovulation failure in mice [194]. However, the research primary focus on single-compound exposures does not really reflect the real-life exposure to complex mixtures of PFAS. Future studies must be designed to reflect the real-life mixtures exposures.

**Figure 4:**
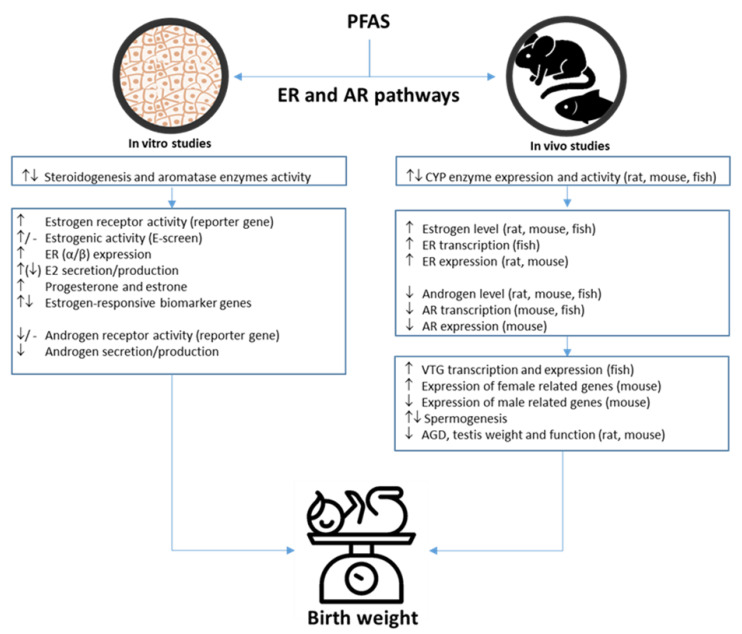
Possible PFAS-induced estrogenic and androgenic effects involved in birth weight. PFAS, per- and polyfluoroalkyl substances; ER, estrogen receptor; AR, androgen receptor; VTG, vitellogenin; E2, estradiol; AGD, anogenital distance.

There is evidence for PFAS affecting ER signaling in humans and animals although it is not consistent [190]. Study reports suggest an ability of PFAS to modulate and/or further activate ER-mediated effects [36,99,104,109,195,196] with some effects only observed in aquatic organisms [106,119,197]. Microarray analyses of human primary hepatocytes confirmed that PFOA activated the ER pathway [131]. The PFAS in general elicits estrogenic effects, mainly mediated via the estrogen receptor. There are indications of anti-androgenic effects as well (e.g., decrease testosterone level) even though only one of six reviewed in vitro studies found significant anti-androgenic through the AR receptor [99]. PFAS may influence human sex hormone biosynthesis, serum, and tissue hormone levels and receptor expression and function and thereby fetal growth (Figure 4). Whether the effect on fetal growth is mediated through the alteration of the sex hormone system is unknown, but a possible mechanism could be by an alteration in the placental development and function. As already mentioned, we previously found that the serum PFAS-induced ER activity was associated with decreased birth weight and length [36].

There are some suggested sex differences in the effects of PFAS on fetal growth—although the data are not consistent. The effects on the sex hormone system might provide possible explanations for the sexual dimorphism to PFAS exposure. Two of the reviewed studies also support the sex differences, as CYP19A expression in zebra fish increased in female gonads and decreased in male gonads [106], and Rosen et al. [112] found decreased expression of male-specific genes and increased expression of female-specific genes after PFAS exposure in mice. Interestingly, the sex-specific results are not only seen for fetal growth, but epidemiological studies found associations between prenatal PFAS exposure and adiposity and overweight for females, but not males, later in life [198,199].

Thyroid hormones are essential for normal fetal growth and development. The fetus is completely reliant on maternal T4 during the first trimester; thereafter, the fetal thyroid gland begins to function [200,201]. At birth, approximately 30% of T4 in cord blood originates from the mother [202]. Thus, there are concerns about the potential effect of in utero PFAS exposure on thyroid hormone homeostasis in pregnant women and their fetuses [203]. Therefore, thyroid hormones are of critical importance to both pregnant women and their offspring. Decreased maternal provision of T4 to the fetus leads to an increased risk of poor cognition, behavior, and growth [204,205,206].

**Figure 5:**
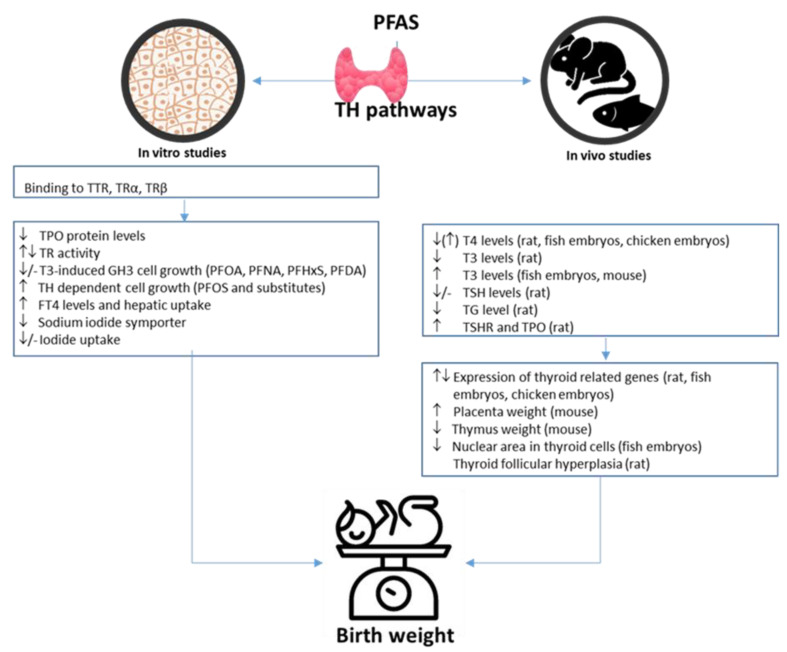
Possible PFAS-induced thyroid effects involved in birth weight. PFAS, per- and polyfluoroalkyl substances; TH, thyroid hormone; TPO, thyroperoxidase; TR, thyroid hormone receptor; T4, thyroxine; T3, triiodothyronine; FT4, free T4; TSH, thyroid-stimulating hormone; TG, thyroglobulin; TSHR, TSH receptor.

The in vitro and in vivo studies evaluated in this review elicited that PFAS can interfere with thyroid hormone levels and functions in synthesis, cell levels, transport, binding to receptor, and receptor function (Figure 5). Several epidemiological studies have investigated the association between PFAS and TSH levels, and the majority of the findings are significant positive [206]. However, only two of the reviewed in vivo studies investigated TSH level, with one finding a decrease in TSH [140] and one finding no effect in rats after PFAS exposure [147]. For T3 and T4, both epidemiological studies [206] and the reviewed in vivo studies generally found inverse associations with PFAS exposure, but the results are conflicting. Overall, this review suggests some evidence for thyroid-disrupting effects in in vitro and animal models, whereas human studies provide some conflicting results. Further research including more longitudinal and long-term follow-up on population studies might give further knowledge about the detailed pathways involved in the impact on fetal growth.

## 5. Strengths and Limitations of the Study

The review provides an overview of different PFAS-affected pathways both at the molecular level and at the functional level (e.g., receptor activity). There are specific differences in human and rodent biology and health outcomes that deserve further investigation. The extent to which results from in vitro studies and in vivo animal studies are transferable to human health needs to be verified and confirmed in further studies. The use of an automatic tool such as AOP-helpFinder to screen and extract information from the literature is an advantage, as the researcher does not have to perform each search independently. It is therefore less time consuming and allows to obtain a good overview of the existing data that have been published. Nevertheless, results obtained by such approach need validation by experts. The present study was limited to exploration of the literature from the *PubMed* database to identify stressor–event linkage. The AOP-helpFinder tool is currently under optimization in order to be able to also decipher relationships between key events, which will allow a more complete exploration of available data and will require less manual curation by experts. Information from various databases will also be screened and added following an integrative systems biology pipeline [48].

## 6. Conclusions and Future Experimental Model Studies

The described PFAS-induced changes in ROS signaling or endocrine system and their respective influence on birth weight appear unrelated. Although interconnection of individual parts, somehow, is evident (e.g., estrogen receptors have been identified as redox sensors [207]), it remains to be elucidated whether and how oxidative stress and/or adipocyte differentiation and/or general endocrine dysfunction (mediated through the estrogenic, androgenic, and/or thyroid hormone systems) interact to affect birth weight.

Our search did not reveal any study testing a model for pregnancy-related diseases such as animal models for SGA [208]. However, it can be assumed that the basic cellular signaling pathways are evolutionarily conserved. In general, AOPs are developed using all available data from different cellular models complemented by animal studies, as data from in vitro studies should be further supported by in vivo animal studies (e.g., [209]). Genes/proteins described in animal studies may be named differently but are mostly functionally analogous to those in humans [210,211].

Experimental studies in cell systems or animals are critical for elucidating the human health effects of PFAS, e.g., on liver, thyroid, and lipid homeostasis. Some effects in cell systems/animals were not identical to those in humans, and new targets were identified, e.g., mammary gland and immune system changes. Long-term exposure to relevant doses of PFAS, e.g., in animal models, could help elucidate PFAS-induced fetal growth restriction. Experimental in vitro and in vivo studies are needed to confirm key molecular events involved in potential novel AOPs. Future studies also need to examine the effects of complex PFAS mixtures to account for real-life exposure. Another future research direction may be to investigate the interactions of PFAS with other chemical/non-chemical stressors.

## Figures and Tables

**Table 1:** Reviews analyzing epidemiological studies for associations between prenatal PFAS exposure and birth weight indices.

Source	Type of Analysis	No of Studies (Publication Date)	Analyte(s)	Surveyed Birth Outcome	Main Results	Conclusions
Bach et al., 2015 [28]	Systematic review	14 (2004–2013)	PFOA, PFOS	Birth weight Low birth weight ^a^ Small for gestational age ^b^ Birth weight z-scores ^c^	PFOA exposure associated with decreased measures of continuous birth weight in all studies at different magnitudes, with many results being statistically insignificant PFOS: no clear trend for effects on birth weight	“The impact on public health is unclear”
Negri et al., 2017 [29]	Systematic review	16 (up to 2015)	PFOA, PFOS	Birth weight	PFOA: −12.8 g/ng/mL (−27.1 g per increase of 1 log_e_ ng/mL) PFOS: −0.92 g/ng/mL (−46.1 g per increase of 1 log_e_ ng/mL)	“…no quantitative toxicological evidence to support the epidemiological association, thus reducing the biological plausibility of a causal relationship”
Govarts et al., 2018 [6]	Pooled analysis	7 birth cohorts 5446 mother–child pairs	PFOA, PFOS	Small for gestational age ^b^	PFOA: Higher levels associated with greater risk of SGA (OR: 1.64) PFOS: Higher levels associated with greater risk of SGA (OR: 1.63) in newborns of mothers who smoked during pregnancy (but decreased risk in newborns of non-smoking mothers (OR: 0.66))	“Prenatal environmental exposure to perfluorinated compounds with endocrine disrupting properties may contribute to the prevalence of SGA. We found indication of effect modification by child’s sex and smoking during pregnancy. The direction of the associations differed by chemical and these effect modifiers, suggesting diverse mechanisms of action and biological pathways”
Dzierlenga et al., 2020 [30]	Random-effects meta-regression	29 (up to 2019)	PFOS	Birth weight	−3.22 g/ng/mL (all) −1.35 g/ng/mL (early group ^d^) −7.17 g/ng/mL (later group ^d^)	“...when blood was drawn at the very beginning of pregnancy, there was essentially no relation of birth weight to PFOS”, “stronger inverse association in Asian studies”, “The evidence was weakly or not supportive of a causal association”
Cao et al., 2021 preprint [31]	Meta-analysis (fixed-effect and random-effect models)	6 (2009–2017)	PFOA, PFOS	Low birth weight ^a^	PFOA: OR = 0.90 PFOS: OR = 1.32 (America: OR = 1.44)	“...study provided a systematic review and meta-analysis evidence for the relationship between maternal PFASs exposure and LBW of offspring through a small number of studies. Researchers should conduct further studies between different regions”
Lee et al., 2021 [32]	Systematic review	90 (2007–2021)	PFOA, PFOS, 11 other PFAS	Birth weight Birth length Ponderal Index ^e^ Gestational age	Most studies suggest that prenatal PFAS exposure (especially long-chain PFAS) may affect fetal growth	“The current epidemiologic evidence has mostly suggested that prenatal PFAS exposures may impair fetal growth... The mechanisms through which PFAS affect early-life physical development in humans remain unclear”

^a^ Low birth weight: birth weight below 2500 g. ^b^ Small for gestational age (SGA): birth weight below the 10th growth percentile for gestational age. ^c^ Birth weight z-scores: birth weight standardized for sex and gestational age; ^d^ “early group”: maternal blood drawn during early pregnancy; “later group”: maternal blood drawn during late pregnancy. ^e^ Ponderal Index: birth weight in relation to birth length.

**Table 2:** Search terms on adverse outcomes and health effects including MIEs and KEs used in AOP helpFinder search.

MeSH Terms	Other Search Terms
Infant, Small for Gestational Age	Amino acids
Infant, Small for Gestational Age/growth and development	Nutrients
Infant, Small for Gestational Age/blood	Glucose
Infant, Small for Gestational Age/metabolism	Fatty acids
Infant, Small for Gestational Age/physiology	Fetal growth restriction
Premature Birth	Intrauterine growth retardation
Pre-Eclampsia	Intrauterine growth restriction
Receptor, Fibroblast Growth Factor, Type 1	Placenta malperfusion
Placenta Diseases	Vascular endothelial growth factor
Placenta Growth Factor	Flt-1
Placenta Growth Factor, PLGF-1 Isoform	Thiol adduct
Receptors, Vascular Endothelial Growth Factor	Thio/seleno-protein
Receptors, Androgen	Oxidative stress
Receptors, Estrogen	DNA polymerase gamma
	Small for Gestational Age
	Small for Gestational Age/growth and development
	Small for Gestational Age/blood
	Small for Gestational Age/metabolism
	Small for Gestational Age/physiology
	Fetal growth restriction
	Intrauterine growth retardation
	Intrauterine growth restriction
	IUGR
	Inhibition Cytochrome P450 enzyme activity
	Inhibition CYP17A1 activity
	Decreased Aromatase mRNA
	Decreased Cyp19a1 mRNA
**Fetal growth**	
AO	Increase, Growth inhibition
AO	Growth, reduction
AO	Decrease, Growth
MIE	Inhibition, VegfR2
KE	Decreased, angiogenesis
KE	Defect of Embryogenesis
KE	Decrease, Growth
KE	Reduction, Progesterone synthesis
**Oxidative stress**	
MIE	Activation, NRF2
KE	ROS formation
KE	Increase, Oxidative Stress
KE	Activation, PMK-1 P38 MAPK
KE	Down Regulation, GSS and GSTs gene
KE	Glutathione synthesis
KE	Glutathione homeostasis
MIE	Thiol group of chemicals interact with sulfhydryl groups of proteins to form thiol adducts
MIE	Inhibition of mitochondrial DNA polymerase gamma
KE	Dysfunction, Mitochondria
MIE	Binding, Thiol/seleno-proteins involved in protection against oxidative stress
**Signaling pathways**	
KE	Activation, AKT2
KE	Activation, HIF-1
KE	Activation, JAK/STAT pathway
KE	Activation, TGF-beta pathway
KE	Activation, JNK
MIE	Wnt ligand stimulation
KE	Inhibition, Wnt pathway
KE	Frizzled activation
KE	Alteration, Wnt pathway
**Endocrine related pathways**	
MIE	Activation, Androgen receptor
MIE	Decreased, Androgen receptor activity
MIE	Activation, Estrogen receptor
KE	Increased, Estrogen receptor activity
KE	Increased, ER activity
KE	Decrease, testosterone synthesis
KE	Decrease, testosterone level
KE	Decrease, dihydrotestosterone level
KE	Decrease, DHT level
KE	Decrease, androgen receptors (AR) activation
KE	Decrease, AR activation
KE	Reduction, 17-OH-pregnenolone conversion in DHEA
KE	Reduction, 17-OH-progesterone conversion in androstenedione
KE	Thyroid hormone disruption
**Others**	
MIE	Inhibition, Cytochrome P450 enzyme (CYP17A1) activity
MIE	Binding of substrate, endocytic receptor
MIE	Inhibition, Aromatase
KE	Decreased, Aromatase (Cyp19a1) mRNA
KE	Perturbation of cholesterol
KE	GSK3beta inactivation
KE	β-catenin activation

Abbreviations: AO, adverse outcome; MIE, molecular initiating event; KE, key event.

**Table 3:** Search terms on PFAS.

PFAS, General Terms	
PFAS, Perfluoroalkyl substances	
Perfluoroalkyl substances	
Perfluoroalkyl acids	
Perfluoroalkyl carboxylates (PFCAs)	
Perfluroalkylated substances	
PFC, Perfluorinated compound	
Perfluorinated sulfonates (PFSAs)	
PFAS compounds *	
PFCAs	
C8	PFOA, perfluorooctanoic acid
C9	PFNA, perfluorononanoic acid
C10	PFDA, perfluorodecanoic acid or perfluoro-n-decanoic acid
PFSAs	
C6	PFHxS, perfluorohexanesulfonic acid or perfluoro-1-hexanesulfonate
C8	PFOS, perfluorooctane sulfonic acid or perfluorooctane sulfonate

* PFAS compounds listed according to functional group (i.e., carboxylated or sulfonated) and carbon chain length (C6–C10).

**Table 4:** In vivo results related to PFAS exposures and oxidative stress.

Publication Information	Study Setup													
Authors	Species	Solvent	Body Weight	Fetal Weight	Offspring Weight	Rep/Dev	Amino acids	Glucose	Lipids	Liver	Oxidative Stress	Lethality	Specific Genes	Other AO
**PFOS**														
Kim et al., 2020 [53]	*C. elegans*					↓	↑↓		↑↓		↑	↑		
Kim et al., 2021 [54]	Drosophila	Aceton	↓			↓			↑↓			↑		
Lee et al., 2015 [55]	Female CD-1 mice	DMSO							↑↓		↑			
Li et al., 2020 [56]	Adult CD-1 mice	DMSO												DNA Methylation
Li et al., 2020 [57]	*C. elegans*	Water							↑↓					
Ortiz-Villanueva et al., 2018 [58]	Zebrafish	DMSO					↑↓							Metabolome
Park et al., 2020 [59]	*Macrophthalmus japonicus crab*													Involvement of MAPK/p38
Qiu et al., 2016 [60]	Male ICR mice	DMSO	–									↑		
Seyoum et al., 2020 [61]	Daphnia	Water	↓			↓			↑↓			↑		
Wan et al., 2020 [62]	CD1 mice	DMSO		↓		↓							SNAT4	
Wang et al., 2020 [63]	*Dugesia japonica*	DMSO									↑		SOD, CAT, GPx1	
Xia et al., 2018 [64]	*Anodonta woodiana*	DMSO										↑		
Yue et al., 2020 [65]	*C. elegans*	Water	↓			↓			↑↓			↑		Metabolism
Zhang et al., 2020 [66]	Manila clam	DMSO									↑↓			Metabolism/Genes
**PFOA**														
Du et al., 2018 [67]	Male Balb/c mice	DMSO	↓					↑	↑			↑		
Guruge et al., 2006 [68]	Male Sprague–Dawley rats		↑↓				↑↓		↑↓				Transcriptome	Gene expression
Kim et al., 2020 [53]	C. elegans					↓	↑↓		↑↓		↑	↑		Metabolism; Lipidomic
Li et al., 2019 [69]	Kunming mice			↓						↑↓	↑	↑		Gene expression
Li et al., 2021 [70]	M. edulis	Water									↑		CAT/SOD/GPx	
Liu et al., 2015 [71]	Male mice	Water				↓					↑			
Liu et al., 1996 [72]	Male rats	unclear	↓							↑				
Salimi et al., 2019 [73]	Mouse		↓								↑			
Seyoum et al., 2020 [61]	Daphnia		–			↓			↑↓			↑		
Wang et al., 2010 [74]	Drosophila		↓		↓	↓								Reduced longevity of males
Xia et al., 2018 [64]	*Anodonta woodiana*	DMSO										↑		
Yan et al., 2015 [75]	Male Balb/c mice	Water						↑↓	↑↓	↑↓			Akt, GSK	
Yang 2010 [76]	*Oryzias latipes*	Water									–		PPR alpha	

Rep/Dev, effects on reproduction and/or development; green cell (↑), increase; orange cell (↓), decrease; blue cell, changes in different directions; grey cell (–), no effect. For more details, see Supplementary Table S1.

**Table 5:** In vitro results related to PFAS-induced oxidative stress and reduced cellular health.

Publication Information	Study Setup									
Authors	Cell System	Species	Reproduction	Amino Acids	Glucose	Lipids/Fats	Oxidative Stress	Cytotoxic/Reduced Cell Number	Spec. Genes	Other AO
**PFOS**										
Chiu et al., 2018 [77]	Not described	Tox-Screening; ToxCast; Tox21					↑			Many different AO
Gorrochategui et al., 2014 [78]	JEG-3	Human						↑		
Gorrochategui et al., 2016 [79]	A6 Kidney Epithelial Cells	Xenopus laevis						↑		
Li et al., 2020 [56]	HTR-8/SVneo	Human						↑		
Reistad et al., 2013 [80]	Cerebellar granule cells	Rat					↑	↑		
Sun et al., 2018 [81]	SH-SY5Y Cell	Human					↑	↑	NRF2, HO-1	
Sun et al., 2019 [82]	SH-SY5Y Cell	Human					↑	↑	JNK-1	
Tang et al., 2017 [83]	ES cell line D3	Mouse				↑	↑	↑	Mfn1, Mfn2, mTOR, RICTOR	Ca2+ flux is impaired
Wang et al., 2015 [84]	HAPI microglial cells	Rat					↑	↑	ERK, JNK, p38	
Wei et al., 2009 [85]	Primary hepatocytes	Gobiocypris rarus (fish)				↑	↑	↑		Gene expression
Xu et al., 2016 [86]	3T3-L1 pre-adipocytes	Mouse			↑	↑↓		↑	NRF2, Lpl, NQo1, PPAR, FABP4	
Zarei et al., 2018 [87]	Lymphocytes	Human					↑	↑		
**PFOA**										
Chiu et al., 2018 [77]	Not described	Tox-Screening; ToxCast; Tox21					↑			Many different AOs
Gorrochategui et al., 2014 [78]	JEG-3	Human						↑		
Gorrochategui et al., 2016 [79]	A6 Kidney Epithelial Cells	Xenopus laevis						↑		
Lu et al., 2016 [88]	Sperm cells	Mouse	↓			↓	↑	↑	FABP3/4/6/KAR/ELOVL5	AKT
Mashayekhi et al., 2015 [89]	Rat mitochondria (liver/brain)	Rat					↑	–		No changes in GSH levels
Reistad et al., 2013 [80]	Cerebellar granule cells	Rat					↑			
Suh et al., 2017 [90]	RIN-m5F cells	Rat					↑	↑		
Tang et al., 2018 [91]	Primary lymphocytes	C. auratus					↑	↑		
Tian et al., 2021 [92]	RAW264.7	Mouse		↑↓		↑↓	↑	↑		
Wei et al., 2009 [85]	Primary hepatocytes	*Gobiocypris rarus* (fish)				↑	↑	–		
**PFNA**										
Gorrochategui et al., 2014 [78]	JEG-3	Human						↑		
Wei et al., 2009 [85]	Primary hepatocytes	*Gobiocypris rarus* (fish)				↑	↑	–		
**PFHxS**										
Gorrochategui et al., 2014 [78]	JEG-3	Human						–		
Lee et al., 2014 [93]	Neuronal cells	Rat					↑	↑		
Lee et al., 2014 [94]	PC12	Rat					↑	↑		
**PFDA**										
Dong et al., 2017 [95]	AGS gastric epithelial cells	Human						↓		
Kleszczyński et al., 2011 [96]	HCT116	Human								Ca ions inside mitochondria
Wei et al., 2009 [85]	Primary hepatocytes	*Gobiocypris rarus* (fish)					↑	–		
Xu et al., 2019 [97]	Hepatic cells	Mouse					↑			DNA Damage
**Mixture**										
Wei et al., 2009 [85]	Primary hepatocytes	*Gobiocypris rarus* (fish)					↑	–		

Green cell (↑), increase; orange cell (↓), decrease; blue cell, changes in different directions; grey cell (–), no effect. For more details, see Supplementary Table S2.

**Table 6:** In vivo results related to PFAS exposures and cellular signaling pathways.

Publication Information	Study Setup		Cellular Signaling				
Authors	Species	Target Tissue	P-AKT (Thr308)	p-AKT (S473)	P38 mRNA	PPARα mRNA	PPARγ mRNA
**PFOS**							
Park et al., 2020 [59]	Crab (*Macrophthalmus japonicus*)	Gill, hepatopancreas			↑		
Qiu et al., 2016 [60]	Mouse (male ICR mice 8 weeks of age)	Testes		↑			
Xu et al., 2016 [86]	Mouse (C57BL/6 mice 10 weeks of age)	Epididymal white adipose tissue					↑
Zhang and Sun et al., 2020 [66]	Clam (*R. philippinarum*)	Hepatopancreas					↑
**PFOA**							
Du et al., 2018 [67]	Mouse (male Balb/c mice 6–7 weeks of age)	Adipose tissue		↓			
Lu et al., 2016 [88]	Mouse (male Balb/c mice 6–8 weeks of age)	Epidymis	↑				
Yan et al., 2015 [75]	Mouse (male Balb/c mice 6–8 weeks of age)	Liver	↑	↑			
Yan et al., 2015 [75]	Mouse (male Balb/c mice 6–8 weeks of age)	Muscle		↑			
Yan et al. 2015 [75]	Mouse (male Balb/c mice 6–8 weeks of age)	White adipose tissue		↓			
Yang 2010 [98]	Fish (male medaka fish)	Liver				↑	

Green cell (↑), increase; orange cell (↓), decrease. For more details, see Supplementary Table S3.

**Table 7:** In vitro results related to PFAS exposures and cellular signaling pathways.

Publication Information	Study Setup		Cellular Signaling			
Authors	Species	Cell Type	P-ERK	P-JNK	P-p38	PPARγ mRNA
**PFOS**						
Qiu et al., 2016 [60]	Mouse (male ICR mice 8 weeks of age)	Primary Sertoli cells			↑	
Sun et al., 2019 [82]	Human	SH-SY5Y (neuroblastoma)		↑		
Wang et al., 2015 [84]	Rat	HAPI (microglia-like cell line)	↑	↑	–	
Xu et al., 2016 [86]	Mouse	Adipocytes derived from 3T3-L1 preadipocyte cell line				↑
**PFHxS**						
Lee et al., 2014 [94]	Rat	PC12 (adrenal gland)	↑	↑	↑	
Lee et al., 2014 [93]	Rat (7-day old Sprague–Dawley rat pups)	Primary cerebella granular cells	↑	↑	↑	

Green cell (↑), increase; grey cell (–), no effect. For more details, see Supplementary Table S3.

**Table 8:** In vivo results related to PFAS exposures and estrogen and androgen pathways.

Publication information	Study Setup	Estrogenic and Androgenic Related Results								
Authors	Species	Estrogen levels	ER Transcription (mRNA)	ER Expression (Protein)	VTG	Testosterone Levels	AR Transcription (mRNA)	AR Expression(protein)	CYP	Other Directly Estrogenic/Androgenic Related Effects
**PFOS**										
Bao et al., 2019 [102]	Female zebrafish	↑ ↓	↑ ↓		↑ ↓					Altered gene expression along the HPGL axis
Bao et al., 2020 [103]	Male zebrafish		↑		↑		↓			↓FSH and LH receptor in gonads, ↓ expression of GnRH, GNRHr, FSH, and LH in brain, impaired sexual behavior
Benninghoff et al., 2011 [104]	Juvenile rainbow trout				–					
Biegel et al., 1995 [105]	Rats (male CD)	↑				↑			↑CYP19	
Chen et al., 2016 [106]	Zebra fish (Post-fertilization)	↑	↑			↓			CYP19A ( ↑ female) / ( ↓ male)	↑ amh (gonad), structural changes in gonads
Du et al., 2013 [107]	Zebrafish embryo		↑ ↓						↓CYP17, CYP19a, CYP19b	
Qu et al., 2016 [108]	Mouse (C57 male)	–	↑ ↓			↓				↓ sperm concentration, vacuolations observed in spermatogonia, spermatocytes and Leydig cells, ↑ incidence of apoptotic cells (testes)
Qiu et al., 2020 [109]	Famale Spague Dawley rat	↑		↑						
Qiu et al., 2021 [110]	Mouse (ICR male)	–				↓				No effect on LH or FSH, ↓ sperm count, damaged testicular interstitium morphology
Rodríguez-Jorquera et al., 2019 [111]	Fathead minnow (*Pimephales promelas*)		↑							
Rosen et al., 2017 [112]	Mouse (wt and ppara-null)									Gene-expression: ↓ male-specific genes, ↑ female-specific genes
Xin et al., 2020 [113]	Zebra fish	↑	↑		↑				↑ CYP19a, ↓ CYP19b	Altered spermgenesis
Xu et al., 2017 [114]	Mouse (-/- and +/+ ERβ)			↑						Only in ERβ +/+ mice: hydropic degeneration and vacuolation in hepatocytes, increase cholesterol and bile acid, altered liver genes.
Zhang and Lu et al., 2020 [115]	Rats (pregnant Sprague-Dawley)					↓			↓CYP11A1, CYP17A1, Hsd17b3	↓ Dhh and SOX9 (sertoli cells), affected proliferation (leydig stem cells)
Zhao et al., 2014 [116]	Rats (pregnant Sprague-Dawley)					↓			↓Cyp11a1 Cyp17a, Hsd3b1	↓ AGD and testicular weights (male pups), impaired fetal Leydig cells, ↓ fetal Leydig cells number
Zhong et al., 2016 [117]	Mouse (C57BL/6)	↑				↓				
**PFOA**										
Benninghoff et al., 2011 [104]	Juvenile rainbow trout				↑					
Lu et al., 2019 [118]	Rat (Sprague-Dawley with eliminated Leydig cells)					↓			↓CYP11A1, CYP17A1	No effect on serum FSH and LH, ↓ expression of Lhcgr, Scarb1, Star, Hsd3b1 and Hsd11b1 in leydig cells, affected proliferation of stem Leydig cells
Qiu et al., 2020 [109]	Female Sprague Dawley rat	↑		↑						
Rosen et al., 2017 [112]	Mouse (wt and ppara-null)									↓ expression of male-specific genes, ↑ expression of female-specific genes
Wei et al., 2007 [119]	Freshwater rare minnow		↑		↑					↓ Degenerating vitellogenic-stage oocytes
Xin et al., 2019 [120]	Zebra fish	↑			↑					
Yao et al., 2014 [121]	Female CD-1 mouse									No effect of ER target genes
Zhao et al., 2010 [122]	Female C57Bl/6 mice	–		↑						↑ serum progesterone, ↑ mammary gland responses to estrogen and progesterone, ↑ liver steroid hormone metabolic enzyme gene expressions, no effect on SHBG
**PFNA**										
Benninghoff et al., 2011 [104]	Juvenile rainbow trout				↑					
Feng et al., 2009 [123]	Rat (Sprague–Dawley male)	↑				↑ ↓				No effect on FSH and LH
Rosen et al., 2017 [112]	Mouse (wt and ppara-null)									↓ expression of male-specific genes, ↑ expression of female-specific genes
Singh et al., 2019 [124]	Mouse (prepubertal Parkers male)								↓CYP11A	
Singh et al., 2019 [125]	Mouse (prepubertal Parkers male)						↓	↓		↓ Impairment in testicular functions, Decreased overall germ cell transformation
**PFHxS**										
Rosen et al., 2017 [112]	Mouse (wt and ppara-null)									↓ expression of male-specific genes, ↑ expression of female-specific genes
**PFDA**										
Benninghoff et al., 2011 [104]	Juvenile rainbow trout				↑					
**Mixture**										
Benninghoff et al., 2011 [104]	Juvenile rainbow trout				↑					
Rodríguez-Jorquera et al., 2019 [111]	Fathead minnow (Pimephales promelas)		↑							

Green cell (↑), increase; orange cell (↓), decrease; blue cell, alteration or change in different directions; grey cell (–), no effect. For more details, see Supplementary Table S4. ER, estrogen receptor; AR, androgen receptor; VTG, vitellogenin; CYP, cytochromes P450; HPGL, hypothalamus–pituitary–gonadal–liver; GnRH, gonadotropin-releasing hormone; GNRHr, gonadotropin-releasing hormone receptor; FSH, follicle-stimulating hormone; LH, luteinizing hormone; Dhh, desert hedgehog.

**Table 9:** In vitro results related to PFAS exposures and estrogen and androgen pathways PFAS-associated thyroid hormone effect.

Authors	Species	Estrogen activity		ERα Expression	ERβ Expression	E2 Secretion/Production	Androgen activity	AR Protein	T Secretion/Production	CYP Enzyme Activities	Other Estrogen-Related Effects
		reporter gene	E-screen								
**PFOS**											
Xin et al., 2020 [113]	Human	↑	↑								
Gogola et al., 2020 [126]	Human					↓					↑2-OHE1/E2 ratio
	Human					↑					↓ 2-OHE1, 16-OHE1, 2OHE1/E2 ratio, 16-OHE1/E2 ratio
Halsne et al., 2016 [127]	Human										Normal acini maturation affected, ER-independent mechanisms to normal development of glandular breast tissue
Xu et al., 2017 [114]	Human				↑						
Benninghoff et al., 2011 [104]	Human	↑									
Maras et al., 2006 [128]	Human		–								Altered expression of estrogen-responsive biomarker genes
Li et al., 2020 [129]	Human	↑									Altered expression of estrogen-responsive biomarker genes
Ishibashi et al., 2008 [130]	Yeast	–									
Behr et al., 2018 [101]	Human	↑	–				–				Increased progesterone and estrone, no effect on estrogen- or androgen-responsive genes,
Du et al., 2013 [107]	Monkey and human	↑				↑	–		↓	Altered gene expression	
Rosen et al., 2017 [112]	Human	↑					–				
Biegel et al., 1995 [105]	Rat					–			↓		
Kjeldsen et al., 2013 [99]	Human and hamster	↑					↓			Aromatase unchanged	
Kang et al., 2016 [100]	Human	↓				↑	–		↓	↑CYP17, 3b-hsd2, cyp19	↑ Estrone
**PFOA**											
Xin et al., 2019 [120]	Human	↑									
Yao et al., 2014 [121]	Human	–									
Gogola et al., 2020 [126]	Human					↓					↑2-OHE1/E2 ratio
	Human					↑					↓2-OHE1, 16-OHE1, 2OHE1/E2 ratio, 16-OHE1/E2 ratio
Halsne et al., 2016 [127]	Human										Normal acini maturation not affected
Benninghoff et al., 2011 [104]	Human	↑									
Maras et al., 2006 [128]	Human		–								Altered expression of estrogen-responsive biomarker genes
Li et al., 2020 [129]	Human	↑									Altered expression of estrogen-responsive biomarker genes
Ishibashi et al., 2008 [130]	Yeast	–									
Behr et al., 2018 [101]	Human	↑	–				–			↑CYP21A2	↑ Estrone, no effect on estrogen- or androgen-responsive genes
Buhrke et al., 2015 [131]	Human			↑↓							
Rosen et al., 2017 [112]	Human	↑					–				
Rosenmai et al., 2013 [132]	Human and hamster					↑↓	–		–	Unchanged CYP11A, CYP17 or CYP21	↑Estrone, ↓ androstenedione, no effect on production of progesterone, 17-OH progesterone, or DHEA
Kjeldsen et al., 2013 [99]	Human and hamster	↑					↓			Unchanged Aromatase	
Kang et al., 2016 [100]	Human	↓				↑	–		↓	↑CYP17, 3b-hsd2, cyp19	↑ Estrone
**PFNA**											
Halsne et al., 2016 [127]	Human										Normal acini maturation affected, ER-independent mechanisms to normal development of glandular breast tissue
Benninghoff et al., 2011 [104]	Human	↑									
Maras et al., 2006 [128]	Human										
Li et al., 2020 [129]	Human	↑									Altered expression of estrogen-responsive biomarker genes
Ishibashi et al., 2008 [130]	Yeast	–									
Rosen et al., 2017 [112]	Human	↑					–				
Kjeldsen et al., 2013 [99]	Human and hamster	–					↓			Unchanged Aromatase	
**PFHxS**											
Li et al., 2020 [129]	Human	↑									Altered expression of estrogen-responsive biomarker genes
Behr et al., 2018 [101]	Human	–	–				–			No effect on steroidogenesis	–: No effect on estrogen- or androgen-responsive genes
Rosen et al., 2017 [112]	Human	↑					–				
Kjeldsen et al., 2013 [99]	Human and hamster	↑					↓			Unchanged Aromatase	
**PFDA**											
Halsne et al., 2016 [127]	Human										Normal acini maturation affected, ER-independent mechanisms to normal development of glandular breast tissue
Benninghoff et al., 2011 [104]	Human	↑									
Li et al., 2020 [129]	Human	↑									Altered expression of estrogen-responsive biomarker genes
Ishibashi et al., 2008 [130]	Yeast	–									
Kjeldsen et al., 2013 [99]	Human and hamster	–					↓			↓ Aromatase	
**MIXTURE**											
Gogola et al., 2020 [126]	Human					↓				↑↓	↑ 2-OHE1/E2 ratio
	Human					↑				–	↓2-OHE1, 16-OHE1, 2OHE1/E2 ratio, 16-OHE1/E2 ratio
Gogola et al., 2020 [133]	Human										
	Human										Effect on IGF1 though ERα
Gogola et al., 2020 [133]	Human			–	–						–: Effects were independent of ER pathway
	Human			–	–						–: Effects were independent of ER pathway
Kjeldsen et al., 2013 [99]	Human and hamster	↑					↓			Unchanged Aromatase	
Dairkee et al., 2018 [134]	Human			↑	↓						

Green cell (↑), increase; orange cell (↓), decrease; blue cell, alteration or change in different directions; grey cell (–), no effect. For more details, see Supplementary Table S5. ER, estrogen receptor; E2, 17-beta-estradiol; AR, androgen receptor; CYP, cytochromes P450; 2-OHE1, 2-Hydroxyestrone; 16-OHE1, 6-hydroxyestrone; IGF1, Insulin-Like Growth Factor I; T, testosterone.

**Table 10:** In vivo results related to PFAS exposures and thyroid hormone pathway.

Authors	Species	Body Weight	Organ Weight	Thyroid Hormone Level			Protein Expression/Level			Thyroid Cell Histology	Gene Expression
				T3	T4	TSH	TG	TSHR	TPO		
**PFOS**											
Chen et al., 2018 [135]	Zebrafish embryos				↓					↓nuclear area	↓ thyroid function-related gene expression
Du et al., 2013 [107]	Zebrafish embryos										↑gene related to early thyroid development (hhex and pax8)
Kim et al., 2011 [136]	Zebrafish embryos	↓length		↑	↓						↓TRα, TRβ, hhex, and pax8
Ren et al., 2015 [137]	Amphibians (X. laevis)										↑TH upregulated genes; ↓TH downregulated genes
Shi et al., 2009 [138]	Zebrafish embryos	↓		↑							alter genes in HPT system (↓TSH, TTR, TRα, ↑TRβ)
Yu et al., 2011 [139]	Adult female Wistar rat			↓	↓						↑hepatic genes related to T4 uptake and regulation
***PFOS potassium salt (PFOS-K)***											
Chang et al., 2008 [140]	Female adult SD rat			↓	↓TT4, transit ↑FT4	↓					
***F-53B (PFOS substitute)***											
Deng et al., 2018 [141]	Zebrafish embryos	↓			↑		↓				↑ttr, ↓tg
Hong et al., 2020 [142]	Adult female SD rat			↓	↓			↑	↑	Thyroid follicular hyperplasia	
**PFOA**											
Blake et al., 2020 [43]	Pregnant CD-1 mice	↓embryo	↑placenta								
Godfrey et al., 2019 [143]	Japanese medaka embryo										↑ thyroid-related genes
Kim et al., 2021 [144]	Zebrafish embryos										↑genes related to activation or metabolism
***HFPO-DA (PFOA substitute)***											
Blake et al., 2020 [43]	Pregnant CD-1 mice		↑placenta		↑placenta						
Conley et al., 2021 [145]	SD rat (dam)	↓pup		↓	↓						
**PFNA**											
Liu et al., 2011 [146]	Zebrafish embryos			↑							alter genes related to TH synthesis and metabolism in F1 larvae
**PFHxS**											
Ramhøj et al., 2020 [147]	Wistar rat (dam and offspring)			↓	↓	–					
Cassone et al., 2012 [148]	Chicken embryos	↓embryo			↓						↑TH-response genes
**PFDA**											
Harris et al., 1989 [149]	Adult female C57BL/6 mice	↓	↓Thymus	↑	↑						

Green cell (↑), increase; orange cell (↓), decrease; blue cell, alteration or change in different directions; grey cell (–), no effect. For more details, see Supplementary Table S6. TTR, transthyretin; TPO, thyroperoxidase; TR, thyroid hormone receptor; TG, thyroglobulin; TR, thyroid hormone receptor; TSH, thyroid-stimulating hormone; TSHR, TSH receptor; TH, thyroid hormone; T4, thyroxine; T3, triiodothyronine; FT4, free T4; FT3, free T3; TT4, total T4; TT3, total T3; hhex, hematopoietically expressed homeobox; pax8, paired box gene 8.

**Table 11:** In vitro results related to PFAS exposures and thyroid hormone pathway.

Authors	Cell Species	Protein				T-Screen		NIS	RAIU	Gene Expression	Molecular Docking
		TTR Binding	TBG	TPO	TR		T4				
**PFOS**											
Ren et al., 2016 [150]	Human	B									
Song et al., 2012 [151]	Human			↓							
Selano et al., 2019 [152]	Rat male						↑FT4, ↑hepatic uptake				
Xin et al., 2018 [153]	Human	B	–		B: TRα, TRβ						
	Human				↑Transactivity						Fit into pocket of TTR and TRs
	Rat					↑Compound alone					
Weiss et al., 2009 [154]	Human	B									
Long et al., 2013 [155]	Rat					↓Compound alone and +T3					
Buckalew et al., 2020 [156]	Rat							↓			
	Human							↓			
Wang et al., 2019 [157]	Human								↓		
Song et al., 2011 [158]	Human			↓							
Du et al., 2013 [107]	Monkey				↓Transactivity +T3						
	Human									Altered steroidogenic genes	
Ren et al., 2015 [137]	Human				B: TRα						Fit into T3-binding pocket of TRα-LBD
	Rat					↑Compound alone and +T3					
***PFOS potassium salt PFOS-K***											
Buckalew et al., 2020 [156]	Rat							↓			
	Human							↓			
Wang et al., 2019 [157]	Human								↓		
***F-53B PFOS substitute***											
Deng et al., 2018 [141]	Rat					↑Compound alone					
**PFOA**											
Ren et al., 2016 [150]	Human	B	–								
Song et al., 2012 [151]	Human			↓							
Selano et al., 2019 [152]	Rat male						↑FT4, ↑hepatic uptake				
Weiss et al., 2009 [154]	Human	B									
Long et al., 2013 [155]	Rat					↓Compound alone					
Buckalew et al., 2020 [156]	Rat							↓			
	Human							↓			
Ren et al., 2015 [137]	Human				B: TRα						Fit into T3-binding pocket of TRα-LBD
	Rat					–					
Kim and Lee et al., 2021 [144]	Rat									*Dio2*↓	
***PFOA-ammonium***											
Buckalew et al., 2020 [156]	Rat										
	Human							↓			
Wang et al., 2019 [157]	Human							↓	–		
**PFNA**											
Ren et al., 2016 [150]	Human	B	–								
Weiss et al., 2009 [154]	Human	B									
Long et al., 2013 [155]	Rat					↓Compound alone and +T3					
Wang et al., 2019 [157]	Human								–		
Ren et al., 2015 [137]	Human				B: TRα						Fit into T3-binding pocket of TRα-LBD
	Rat					–					
**PFHxS**											
Ren et al., 2016 [150]	Human	B	–								
Weiss et al., 2009 [154]	Human	B									
Long et al., 2013 [155]	Rat					↓Compound alone and +T3					
Ren et al., 2015 [137]	Human				B: TRα weakly						Fit into T3-binding pocket of TRα-LBD
	Rat					–					
***PFHxS potassium PFHxS-K***											
Buckalew et al., 2020 [156]	Rat							↓			
	Human							↓			
**PFDA**											
Long et al., 2013 [155]	Rat					↓Compound alone					
Wang et al., 2019 [157]	Human								–		
Ren et al., 2015 [137]	Human				B: TRα						Fit into T3-binding pocket of TRα-LBD
	Rat					–			–		
Ren et al., 2016 [150]	Human	B	–								

Green cell (↑), increase; orange cell (↓), decrease; blue cell, alteration or change in different directions; grey cell (–), no effect; purple cell (B), binding to the receptor. For more details, see Supplementary Table S7. TTR, transthyretin; TPO, thyroperoxidase; TR, thyroid hormone receptor; TG, thyroglobulin; TR, thyroid hormone receptor; TSH, thyroid-stimulating hormone; TSHR, TSH receptor; TH, thyroid hormone; T4, thyroxine; T3, triiodothyronine; FT4, free T4; FT3, free T3; TT4, total T4; TT3, total T3; NIS, sodium iodide symporter; RAIU, radioactive iodide uptake; LBD, ligand binding domain; TH, thyroid hormone.

## Data Availability

Not applicable.

## Supplementary Materials

The following supporting information can be downloaded at: https://www.mdpi.com/article/10.3390/toxics10110684/s1, Table S1: In vivo results on PFAS, oxidative stress, and other adverse outcomes; Table S2: In vitro results on PFAS- induced oxidative stress and cellular health; Table S3: In vivo and in vitro results related to PFAS exposures and cellular signaling pathways; Table S4: In vivo results related to estrogen and androgen pathways; Table S5: In vitro results related to estrogen and androgen pathways; Table S6: In vivo studies related to thyroid hormone pathways; Table S7: In vitro studies related to thyroid hormone pathways.
